# Treatment of ruptured intracranial aneurysms yesterday and now

**DOI:** 10.1371/journal.pone.0172837

**Published:** 2017-03-03

**Authors:** Alexander Hammer, Anahi Steiner, Ghassan Kerry, Gholamreza Ranaie, Ingrid Baer, Christian M. Hammer, Stefan Kunze, Hans-Herbert Steiner

**Affiliations:** 1 Department of Neurosurgery, Paracelsus Medical University, Nuremberg, Bavaria, Germany; 2 Institute of Radiology and Neuroradiology, Klinikum Nuremberg, Nuremberg, Bavaria, Germany; 3 Department of Anatomy 2, University of Erlangen-Nuremberg, Universitätsstraße 19, Erlangen, Bavaria, Germany; 4 Department of Neurosurgery, University of Heidelberg, Heidelberg, Baden-Wuerttemberg, Germany; Stellenbosch University Faculty of Medicine and Health Sciences, SOUTH AFRICA

## Abstract

**Objective:**

This prospective study is designed to detect changes in the treatment of ruptured intracranial aneurysms over a period of 17 years.

**Methods:**

We compared 361 treated cases of aneurysm occlusion after subarachnoid hemorrhage from 1997 to 2003 with 281 cases from 2006 to 2014. Specialists of neuroradiology and vascular neurosurgery decided over the modality assignment. We established a prospective data acquisition in both groups to detect significant differences within a follow-up time of one year. With this setting we evaluated the treatment methods over time and compared endovascular with microsurgical treatment.

**Results:**

When compared to the earlier group, microsurgical treatment was less frequently chosen in the more recent collective because of neck-configuration. Endovascular treatment was chosen more frequently over time (31.9% versus 48.8%). Occurrence of initial symptomatic ischemic stroke was significantly lower in the clipping group compared to the endovascular group and remained stable over time. The number of reinterventions due to refilled treated aneurysms significantly decreased in the endovascular group at one-year follow-up, but the significantly better occlusion- and reintervention-rate of the microsurgical group persisted. The rebleeding rate in the endovascular group at one year follow-up decreased from 6.1% to 2.2% and showed no statistically significant difference to the microsurgical group, anymore (endovascular 2.2% versus microsurgical 0.0%, p = 0.11).

**Conclusion:**

Microsurgical clipping still has some advantages, however endovascular treatment is improving rapidly.

## Introduction

Despite continuous progress in the development of more efficient and safer techniques to treat ruptured intracranial aneurysms, subarachnoid hemorrhage (SAH) and the occlusion of aneurysms still remains a challenge for all treating specialists. The overall incidence of SAH worldwide is currently 9 per 100000, but depends on age, sex and region. Thus, rates are higher in Japan (22.7 per 100000) and in Finland (19.7 per 100000) and increase linearly with age [1]. Women suffer 1.2 times more often from SAH than men and the median age of onset at the first SAH is 50–60 years [1, 2]. International guidelines with comprehensive recommendations for the diagnosis and treatment of aneurysmal subarachnoid hemorrhage and the occlusion of ruptured intracranial aneurysms were published by the American Heart Association / American Stroke Association and the European Stroke Organization [2, 3]. The choice of intervention (microsurgical clipping versus endovascular treatment) should be made by vascular specialists of neurosurgery and neuroradiology. It is dependent on factors such as configuration, location and size of the aneurysm as well as age, comorbidities and patient preference [2]. The International Subarachnoid Aneurysm Trial (ISAT) advises to chose endovascular coil embolization if ruptured aneurysms can be treated equally with both modalities [4–6]. Furthermore, aneurysms with a small aneurysm neck and a posterior location should be treated by endovascular coiling [2]. Also, elderly good-grade patients suffering from SAH seem to benefit from the endovascular option, with the exception of those who display ruptured middle cerebral artery aneurysms [7]. Factors promoting microsurgical clipping are aneurysms with unfavorable neck configuration, branching vessels out of the aneurysm sack, middle cerebral artery aneurysms, pericallosal aneurysms or patients with intracerebral hematoma [2].

In the present study modality assignment was basically in accordance with these mentioned principles of the European Stroke Organisation. Before beginning with data acquisition we determined those criteria under consideration of the treating doctors’ expert opinion. The choice of intervention always represented an individual decision in consent with specialists of neuroradiology and vascular neurosurgery and had been classified by the developed predetermined criteria. According to the recent literature on new techniques and modalities in the treatment of ruptured intracranial aneurysms, assessment criteria seem to have changed, resulting in endovascular treatment apparently being more frequently chosen. This study was designed to reflect these hypothetical changes over time and to compare the chronological development of safety and efficacy in both endovascular and microsurgical treatment of ruptured aneurysms, following the defined modality assignment criteria.

## Material and methods

Data and methods pertaining to this study were already used for an evaluation of efficacy and safety of treatment of intracranial aneurysms from 1997 to 2014 within the scope of the comparison of the two treatment methods, microsurgical clipping and coil embolization [8]. The criteria of allocation, which were observed between 1997 and 2014, allowed us to deliver as a core message that a further limitation of complications in the microsurgical arm is possible [8].

These criteria were applied in this study. In contrast to the above-mentionend paper, we evaluated long-term changes of the outcome of the treatment methods in the present study. In order to ensure a more balanced comparison of the endovascular coiling modality, we relinquished the small subgroup of cases of aneurysmal SAH treated by Flow-Diverters, WEB-Devices and Stent-assisted Coiling procedures in the recent collective. Totally, 642 cases of ruptured intracranial aneurysms with consecutive subarachnoid hemorrhage were recorded and analyzed.

Based on the period of time the patients were treated in, they were divided into two groups–an earlier one and a later one. The earlier group comprised 361 treated cases of aneurysm occlusion after subarachnoid hemorrhage from 1997 to 2003 (115 cases of endovascular treatment, 246 cases treated by microsurgical clipping) from Heidelberg (Department of Neurosurgery, University of Heidelberg, Germany). It was compared with a later group composed of 281 cases taken from the periods 2006–2009 and 2012–2014 (137 cases of endovascular treatment, 144 cases treated by microsurgical clipping) at the Department of Neurosurgery, Nuremberg (Paracelsus Medical University, Germany). As in our previously published paper [8], we did not collect data between 2004 and 2005 and between 2010 and 2011 to avoid bias regarding continuity of expert evaluation, decision and treatment of ruptured intracranial aneurysms. In these time intervals we were not able to treat ruptured aneurysms with the high level of experience, competence and treatment skills as from 1997 to 2003 and from 2006 to 2009 and 2012 to 2014. Expert treatment was guaranteed by specialized vascular neurosurgeons and endovascular specialists.

In this observational study and in the previously published observational study [8], we analyzed the common decision process between neurosurgery and neuroradiology before treatment of ruptured intracranial aneurysms. The decision of neuroradiologists and neurosurgeons as to the allocation of the patients to the endovascular or microsurgical treatment arm was part of the standard care of the patients and without any regard to the study. An approval for this setting was obtained by our local ethics commission (Ethik-Kommission der Bayerischen Landesärztekammer). Our review board „Bayerische Landesärztekammer”waived a patient informed consent, as the data are fully anonymised. Nevertheless informed consent of the patients or their relatives was always obtained verbally during the initial hospital stay or during the telephone interview in the follow up. The half year and one year follow-up telephone interview was executed by Gholamreza Ranaie, Hans-Herbert Steiner and Anahi Steiner (Department of Neurosurgery, Paracelsus Medical University, Nuremberg). The clinical follow up procedure (revaluation of data like clinically apparent strokes) was part of the study and not part of standard care. Initiation of further control diagnostic imaging was conducted according to the recommendations of the specialists of neurosurgery or neuroradiology as part of the standard care and not as part of the study. Patients who disagreed in any form were excluded. We recorded the approval of the participants within our database. The study has been performed in accordance with the ethical standards laid down in the 1964 declaration of Helsinki and its later amendments.

Modality assignment represented an individual decision in consent with specialists of neuroradiology and vascular neurosurgery after critical review of the morphological and topographical aneurysm data and further clinical aspects. The microsurgical clipping group had been classified according to predetermined criteria concerning refusal of endovascular treatment. These criteria were in line with the guidelines of the European Stroke Organisation (Table 1) and also were applied in the paper “Efficacy and Safety of Treatment of Intracranial Aneurysms” [8]. All data were generated with substantial participation and under significant supervision of the senior author (H.-H. Steiner). The primary objective was to document the appearance of clinically apparent permanent ischemic stroke, the rebleeding rate and reinterventions at one year as well as the initial occlusion status and the direct mortality during initial hospital stay and the rate of complete occlusion with all available imaging data over time [8].

**Table 1:** Exclusion criteria for endovascular treatment. Fisher-exact tests and chi-square tests examine the 0-Hypothesis H_0_ that there is no difference regarding the exclusion criteria of endovascular treatment between the two chronological groups (1997 to 2003 versus 2006 to 2009 and 2012 to 2014). Special configurations of the aneurysms and clinical factors affected the specialists’ decision of the choice of treatment and have been documented as "no specified reasons."

Exclusion criteria	1997–2003	2006–2009 and 2012–2014	p
Dome–neck ratio <1.5	52 (21.1%)	14 (9.7%)	0.0037
Neck width	55 (22.4%)	9 (6.3%)	< 0.0001
Endovascular approach not feasable	33 (13.4%)	7 (4.9%)	0.0087
Far distal aneurysm	35 (14.2%)	55 (38.2%)	< 0.0001
Intracranial haematoma	22 (8.9%)	29 (20.1%)	0.0015
Aneurysm causing mass effect	3 (1.2%)	2 (1.4%)	1.0
Patient refused	1 (0.4%)	1 (0.7%)	1.0
No specified reasons	38 (15.4%)	21 (14.6%)	0.82
Coil placement failed or occlusion incomplete	7 (2.8%)	6 (4.2%)	0.56
Overall	246 (100%)	144 (100%)	n/a

Within 48 hours after the clinical event patient data (clinical course, diagnostic imaging), decisions and statements of a daily interdisciplinary conference of neuroradiologists and neurosurgeons were recorded. We established a half-year and one-year follow-up (telephone interview) to re-evaluate the occurrence of the clinical symptomatic ischemic stroke status, occlusion status and occurrence of rebleeding.

Criteria for study inclusion were the same as in our previously published paper [8]:

1. Time between sudden clinical event and treatment < 48 hours.
2. Informed consent from the patient, the patient’s relative or the patient’s caretaker.
3. Verification of SAH via cranial CT or lumbar puncture and verification of an associated intracranial aneurysm diagnosed in most cases by digital subtraction angiography, at least by CT angiography if an immediate operation had to be performed.
4. Patient survival until the ruptured aneurysm had been treated by one intervention modality. Patients with infaust prognosis had been excluded.

In cases of insufficient occlusion attempts of one modality crossing over was possible. This was only realized, however, from the endovascular group to the microsurgical group (Table 1: “Coil placement failed or incomplete occlusion”). This resulted in assignment to both treatment arms. However, failed occlusion before crossing over was recorded as “Incomplete occlusion” for the attempting treatment modality.

In the early group from 1997 to 2003, the endovascular and microsurgical subgroups were matched regarding poor WFNS-grade (World Federation of Neurosurgical Societies), sex, age and size of aneurysm. The median age at admission was 52.2 years in the endovascular group versus 52.1 years in the microsurgical group. Mean aneurysm size was 9.1 millimeters (mm) in the endovascular group and 8.9 mm in the microsurgical group. Endovascular treatment focused on aneurysms of the posterior circulation, aneurysms of the anterior cerebral artery and anterior communicating artery, while in the microsurgical group usually middle cerebral artery aneurysms and aneurysms of the internal carotid artery had been occluded. The endovascular group consisted of a significantly higher proportion of WFNS°III patients when compared to the microsurgical clipping group. The surgical group had a significantly higher proportion of WFNS°I-II patients in the older collective. For an overview of the previously mentioned data see Table 2.

**Table 2:** Characteristics of the included early patient collective (1997 to 2003). ACA: Anterior communicating artery, Anterior cerebral artery; ICA: Internal carotid artery; MCA: Middle cerebral artery; VA: Vertebral artery; BA: Basilar artery. Fisher-exact tests and Chi-Square tests examine the 0-Hypothesis H_0_ that there is no difference between the intervention modalities (endovascular group versus microsurgical group) regarding the ratios of the specific patient characteristics (e. g. ratio “WFNS°I-II” to “not WFNS°I-II” in the endovascular group versus ratio “WFNS°I-II” to “not WFNS°I-II” in the microsurgical group).

1997–2003	Endovascular	Microsurgical	OR	95% CI	p
Overall n = 361	115	246			
WFNS Grade					
WFNS I-II	48 (41.7%)	137 (55.7%)	0.57	0.36–0.89	0.013
WFNS III	38 (33.0%)	50 (20.3%)	1.93	1.18–3.18	0.0087
WFNS IV-V	29 (25.2%)	59 (24.0%)	1.07	0.64–1.78	0.81
Sex					
m	49 (42.6%)	91 (37.0%)	1.26	0.81–1.99	0.31
w	66 (57.4%)	155 (63.0%)	0.79	0.50–1.24	0.31
Location					
ACA	59 (51.3%)	73 (29.7%)	2.5	1.58–3.94	< 0.0001
ICA	28 (24.3%)	99 (40.2%)	0.48	0.29–0.79	0.0032
MCA	2 (1.7%)	65 (26.4%)	0.049	0.012–0.21	< 0.0001
VA/BA	26 (22.6%)	9 (3.7%)	7.69	3.47–17.06	< 0.0001

In the recent group from 2006 to 2009 and 2012–2014, the endovascular and microsurgical modality subgroups were matched regarding good, intermediate and poor WFNS-grade, sex, age and size of aneurysm. The median age at admission was 53.3 years in the endovascular group versus 52.1 years in the microsurgical group. Mean aneurysm size was 7.7 mm in the endovascular group and 8.7 mm in the microsurgical group. In the endovascular treatment group significantly more aneurysms of the posterior circulation, aneurysms of the anterior cerebral artery and anterior communicating artery, and aneurysms of the internal carotid artery had been occluded. In the microsurgical group usually middle cerebral artery aneurysms had been treated. For an overview of the previously mentioned data see Table 3.

**Table 3:** Characteristics of the included recent patient data (2006 to 2009 and 2012 to 2014). ACA: Anterior communicating artery, Anterior cerebral artery; ICA: Internal carotid artery; MCA: Middle cerebral artery; VA: Vertebral artery; BA: Basilar artery. Fisher-exact tests and Chi-Square tests examine the 0-Hypothesis H_0_ that there is no difference between the intervention modalities (endovascular treatment group versus microsurgical treatment group) regarding the ratios of the specific patient characteristics (e. g. ratio “WFNS°I-II” to “not WFNS°I-II” in the endovascular group versus ratio “WFNS°I-II” to “not WFNS°I-II” in the microsurgical group).

2006–2009 and 2012–2014	Endovascular	Microsurgical	OR	95% CI	p
Overall n = 281	137	144			
WFNS Grade					
WFNS I-II	69 (50.4%)	73 (50.7%)	0.99	0.62–1.58	1.00
WFNS III	17 (12.4%)	24 (16.7%)	0.71	0.36–1.39	0.31
WFNS IV-V	51 (37.2%)	47 (32.6%)	1.22	0.75–2.00	0.42
Sex					
m	58 (42.3%)	49 (34.0%)	1.42	0.88–2.31	0.15
w	79 (57.7%)	95 (66.0%)	0.70	0.43–1.14	0.15
Location					
ACA	69 (50.4%)	36 (25.0%)	3.04	1.84–5.04	< 0.0001
ICA	39 (28.5%)	20 (13.9%)	2.47	1.35–4.50	0.0027
MCA	7 (5.1%)	84 (58.3%)	0.039	0.017–0.088	< 0.0001
VA/BA	22 (16.1%)	4 (2.8%)	6.70	2.24–19.99	0.00012

Postinterventional diagnostic imaging for evaluation of symptomatic ischemic stroke, occlusion rate and rebleedings had been discussed and consented in a daily interdisciplinary conference of neuroradiologists and neurosurgeons. In the microsurgical group the occlusion rate had been inquired from the treating surgeon immediately after operation or evaluated from postoperative angiographical data, if available. Ischemic stroke had been assigned to the intervention if it had been clinically apparent and if there had been distinct morphological findings within the first 3 days after the procedure in the diagnostic imaging. “Symptomatic ischemic stroke” was defined as a clinically apparent stroke after procedure and “permanent ischemic stroke” as a symptomatic ischemic stroke which persisted clinically at the time of half-year and one-year follow-up.

Results of data acquisition regarding the appearance of clinically apparent permanent ischemic stroke, the rebleeding rate, and reinterventions refer to the interval between initial hospital stay and one-year follow-up. Direct mortality has been recorded during the initial hospital stay. The rate of complete occlusion was evaluated with available imaging data at an average of 13.1 months in the recent group and 22.3 months in the early group. The decrease of the time interval of imaging follow-up after coiling in the recent group (13.1 months) is probably the consequence of long-term results of the occlusion status of endovascular treated aneurysms [9, 10]. Sprengers et al. stated that intracranial aneurysms with adequate occlusion at 6 months after coiling have a low reopening rate needing retreatment after 5 years and, furthermore, that adequately occluded aneurysms at 6 months might not need prolonged imaging follow-up [9]. Moreover, long-term follow-up results of ISAT exhibit a very low rebleeding rate of treated aneurysms [5].

Intensive care did not differ in the two groups (endovascular versus microsurgical), especially in terms of treatment of vasospasm, hydrocephalus, cerebral edema and other complications like pneumonia, diabetes insipidus, sepsis etc.

Statistical analysis was performed using the Microsoft Excel 2007 Software (Microsoft Corporation, Redmond, Washington, USA). Chi-square was calculated only if all cell frequencies of the 2 x 2 contingency tables were equal to or greater than 10. The Fisher exact probability test was applied if the sample size consisted at least of one small sample of less than 10 in the 2 x 2 contingency tables. Fisher-exact tests and Chi-Square tests examine the 0-Hypothesis H_0_ within one chronological group that there is no difference regarding the patient characteristics in the material and methods section (Tables 2 and 3) and the efficacy and safety in the results section (Tables 4 and 5) of the two treatment modalities. Furthermore Fisher-exact tests and Chi-square tests examine the 0-Hypothesis H_0_ that there is no difference regarding the exclusion criteria of endovascular treatment in the methods section (Table 1) and the efficacy and safety of the two chronological groups of one treatment modality in the results section (Tables 6 and 7).

## Results

### Exclusion criteria for endovascular treatment and ratio of endovascular versus microsurgical treatment

We observed a significant decrease of cases of aneurysm neck configuration which led to an exclusion from endovascular treatment (Table 1). Dome-neck ratio < 1.5 was a reason for exclusion in 21.1% (52 of 246) of the cases from 1997 to 2003, while in the recent group only 9.7% (14 of 144) were excluded due to this criterion (p = 0.0037). Exclusion because of neck width decreased significantly from 22.4% (55 of 246) to 6.3% (9 of 144) (p < 0.0001). In contrast the proportion of cases of intracranial hematoma and far distal aneurysms which were assigned to the microsurgical treatment increased significantly from 8.9% (22 of 246) to 20.1% (29 of 144) (p = 0.0015) and from 14.2% (35 of 246) to 38.2% (55 of 144) (p < 0.0001), respectively. The total ratio of endovascular treatment versus microsurgical treatment changed significantly towards a more frequent selection of the endovascular procedure (2006–2009 and 2012–2014) in comparison to the earlier group. (endovascular: 31.9% to 48.8%; OR 0.49; 95% CI 0.36–0.68; p < 0.0001; microsurgical: 68.1% to 51.2%; OR 2.04; 95% CI 1.47–2.81) (Tables 6 and 7).

### Endovascular treatment versus microsurgical treatment in the early (1997–2003) and late (2006–2009 and 2012–2014) group

In the collective of 1997 through 2003 there was a significantly lower rate of absolute symptomatic ischemic stroke (15.7% versus 5.3%; OR 3.33; 95% CI 1.57–7.05; p = 0.0011) and permanent symptomatic ischemic stroke (13.9% versus 4.5%; OR 3.45; 95% CI 1.55–7.71; p = 0.0015) in the microsurgical group compared to the endovascular group (Table 4). Furthermore, the microsurgical clipping procedure was superior regarding the occlusion of ruptured aneurysms. Initial subtotal occlusion was stated in 1.6% percent of the microsurgical cases versus 19.1% of the endovascular cases (OR 14.31; 95% CI 4.80–42.65; p < 0.0001). Initial incomplete occlusion was objectifiable in the endovascular group only (7.0%; p < 0.0001). Complete occlusion was seen in the follow-up diagnostic imaging after 22.3 months in 65 of 102 cases (63.7%) in the endovascular group and in 84 of 85 cases (98.8%) in the microsurgical group (OR 0.021; 95% CI 0.0028–0.16; p < 0.0001). The rebleeding rate at one year was significantly higher in the endovascular group when compared to the microsurgical group (7 of 115 versus 1 of 246; 6.1% versus 0.4%; OR 15.88; 1.93–130.65; p = 0.0017). There had been 16 reinterventions in the endovascular group (13.9%), but no reinterventions in the microsurgical group (p < 0.0001). Direct mortality was not significantly different between the two groups (endovascular 2.6% versus microsurgical 1.2%; p = 0.39).

**Table 4:** Efficacy and safety of the treatment modalities from 1997 to 2003. Fisher-exact tests and Chi-Square tests examine the 0-Hypothesis H_0_ that there is no difference regarding the rates of symptomatic ischemic stroke, occlusion rate, rebleeding, direct mortality and reinterventions between the two treatment modalities (endovascular group versus microsurgical group).

1997–2003	Endovascular	Microsurgical	OR	95% CI	p
Overall n = 361	115	246			
Initial symptomatic ischemic stroke	18 (15.7%)	13 (5.3%)	3.33	1.57–7.05	0.0011
Permanent symptomatic ischemic stroke (one-year follow-up)	16 (13.9%)	11 (4.5%)	3.45	1.55–7.71	0.0015
Initial subtotal occlusion	22 (19.1%)	4 (1.6%)	14.31	4.80–42.65	< 0.0001
Initial incomplete occlusion	8 (7.0%)	0 (0.0%)	n/a	n/a	< 0.0001
Complete occlussion (22.3 months)	65 of 102 (63.7%)	84 of 85 (98.8%)	0.021	0.0028–0.16	< 0.0001
Re-bleeding (one-year follow-up)	7 (6 early) (6.1%)	1 (1 early) (0.4%)	15.88	1.93–130.65	0.0017
Direct mortality	3 (2.6%)	3 (1.2%)	2.17	0.43–10.92	0.39
Reintervention (one-year follow-up)	16 (7 microsurgical clipping) (13.9%)	0 (0.0%)	n/a	n/a	< 0.0001

In the collective of 2006–2009 and 2012–2014 there was also a lower rate of symptomatic ischemic stroke in the microsurgical clipping group compared to the endovascular group (endovascular group: 27 of 137; 19.7%—microsurgical group: 13 of 144; 9.0%; OR 2.47; 95% CI 1.22–5.02; p = 0.010) and a significantly lower rate of permanent symptomatic ischemic stroke (Endovascular group: 21 of 137; 15.3%—Microsurgical group: 11 of 144; 7.6%; OR 2.19; 95% CI 1.01–4.73; p = 0.043)(Table 5). The significantly higher occlusion rate of the microsurgical clipping group remained stable (initial subtotal occlusion: endovascular 20 of 137; 14.6% versus microsurgical 3 of 144; 2.1%; p = 0.00012; initial incomplete occlusion: Endovascular 9 of 137; 6.6% versus Microsurgical 1 of 144; 0.7%; p = 0.0089; complete occlusion after 13.1 months: endovascular 73 of 114; 64.0% versus microsurgical 30 of 34; 88.2%; p = 0.0071) and also the reintervention rate remained significantly lower (endovascular 7 of 137; 5.1% versus microsurgical 0 of 144; 0.0%; p = 0.0060)(Table 5).

**Table 5:** Efficacy and safety of the treatment modalities from 2006 to 2009 and 2012 to 2014. Fisher-exact tests and Chi-Square tests examine the 0-Hypothesis H_0_ that there is no difference regarding the rates of symptomatic ischemic stroke, occlusion rate, rebleeding, direct mortality and reinterventions between the two treatment modalities (endovascular group versus microsurgical group).

2006–2009 and 2012–2014	Endovascular	Microsurgical	OR	95% CI	p
Overall n = 281	137	144			
Initial symptomatic ischemic stroke	27 (19.7%)	13 (9.0%)	2.47	1.22–5.02	0.010
Permanent symptomatic ischemic stroke (one-year follow-up)	21 (15.3%)	11 (7.6%)	2.19	1.01–4.73	0.043
Initial subtotal occlusion	20 (14.6%)	3 (2.1%)	8.03	2.33–27.71	0.00012
Initial incomplete occlusion	9 (6.6%)	1 (0.7%)	10.05	1.26–80.46	0.0089
Complete occlussion (13.1 months)	73 of 114 (64.0%)	30 of 34 (88.2%)	0.24	0.078–0.72	0.0071
Re-bleeding (one-year follow-up)	3 (3 early) (2.2%)	0 (0.0%)	n/a	n/a	0.11
Direct mortality	4 (2.9%)	1 (0.7%)	4.30	0.47–38.97	0.20
Reintervention (one-year follow-up)	7 (1 microsurgical clipping) (5.1%)	0 (0.0%)	n/a	n/a	0.0060

Interestingly, the rebleeding rate at one-year follow-up in the endovascular group decreased from 6.1% to 2.2% and was no longer significantly different compared to the microsurgical clipping group (endovascular 3 of 137; 2.2% versus microsurgical 0 of 144; 0.0%, p = 0.11). Still, there was no significant difference regarding the direct mortality rate of the two procedures (endovascular group: 4 of 137; 2.9%; microsurgical group: 1 of 144; 0.7%; OR 4.30; 95% CI 0.47–38.97; p = 0.20).

### Modality changes over time

Comparison of the older group with the recent group, revealed a significant decrease in the reintervention rate of endovascular treatment of ruptured intracranial aneurysms. It decreased from 13.9% (16 of 115) to 5.1% (7 of 137) (OR 3.00; 95% CI 1.19–7.58; p = 0.017) (Table 6). The complete occlusion rate of the microsurgical treatment decreased from 98.8% (84 of 85) to 88.2% (30 of 34) (OR 11.2; 1.20–104.23; p = 0.023) (Table 7).

**Table 6:** Comparison of the efficacy and safety of the chronological groups (1997–2003 versus 2006 to 2009 and 2012 to 2014) regarding the endovascular procedure. Chi-Square tests examine the 0-Hypothesis H_0_ that there is no difference regarding the rate of endovascular cases (n = 115) in the comprehensive group of 1997–2003 (n = 361) compared to the rate of endovascular cases (n = 137) in the comprehensive recent coiling group (n = 281) (coiling 2006–2009 and 2012–2014). Moreover Fisher-exact tests and Chi-Square tests examine the 0-Hypothesis H_0_ that there is no difference regarding the rates of symptomatic ischemic stroke, occlusion rate, rebleeding, direct mortality and reinterventions between the two chronological groups (“Endovascular 1997–2003” versus “Endovascular 2006–2009 and 2012–2014”).

Endovascular treatment	Endovascular 1997–2003	Endovascular 2006–2009 and 2012–2014	OR	95% CI	p
Overall n = 252	115 of 361 (31.9%)	137 of 281 (48.8%)	0.49	0.36–0.68	< 0.0001
Initial symptomatic ischemic stroke	18 (15.7%)	27 (19.7%)	0.76	0.39–1.46	0.40
Permanent symptomatic ischemic stroke (one-year follow-up)	16 (13.9%)	21 (15.3%)	0.89	0.44–1.80	0.75
Initial subtotal occlusion	22 (19.1%)	20 (14.6%)	1.38	0.71–2.69	0.34
Initial incomplete occlusion	8 (7.0%)	9 (6.6%)	1.06	0.40–2.85	1.00
Complete occlussion	65 of 102 (22.3 months) (63.7%)	73 of 114 (13.1 months) (64.0%)	0.99	0.57–1.72	1.00
Re-bleeding (one-year follow-up)	7 (6 early) (6.1%)	3 (3 early) (2.2%)	2.90	0.73–11.46	0.19
Direct mortality	3 (2.6%)	4 (2.9%)	0.89	0.20–4.06	1.00
Reintervention (one-year follow-up)	16 (7 microsurgical clipping) (13.9%)	7 (1 microsurgical clipping) (5.1%)	3.00	1.19–7.58	0.017

**Table 7:** Comparison of the efficacy and safety of the chronological groups (1997–2003 versus 2006 to 2009 and 2012 to 2014) regarding the microsurgical procedure. Chi-Square tests examine the 0-Hypothesis H_0_ that there is no difference regarding the rate of clipped cases (n = 246) in the comprehensive group of 1997–2003 (n = 361) compared to the rate of clipped cases (n = 144) in the comprehensive recent microsurgical clipping group (n = 281) (microsurgical 2006–2009 and 2012–2014). Moreover Fisher-exact tests and Chi-Square tests examine the 0-Hypothesis H_0_ that there is no difference regarding the rates of symptomatic ischemic stroke, occlusion rate, rebleeding, direct mortality and reinterventions between the two chronological groups (“Microsurgical 1997–2003” versus “Microsurgical 2006–2009 and 2012–2014”).

Microsurgical clipping	Microsurgical 1997–2003	Microsurgical 2006–2009 and 2012–2014	OR	95% CI	p
Overall n = 390	246 of 361 (68.1%)	144 of 281 (51.2%)	2.04	1.47–2.81	< 0.0001
Initial symptomatic ischemic stroke	13 (5.3%)	13 (9.0%)	0.56	0.25–1.25	0.15
Permanent symptomatic ischemic stroke (one-year follow-up)	11 (4.5%)	11 (7.6%)	0.57	0.24–1.34	0.19
Initial subtotal occlusion	4 (1.6%)	3 (2.1%)	0.78	0.17–3.52	1.00
Initial incomplete occlusion	0 (0.0%)	1 (0.7%)	n/a	n/a	0.37
Complete occlussion	84 of 85 (22.3 months) (98.8%)	30 of 34 (12.8 months) (88.2%)	11.2	1.20–104.23	0.023
Re-bleeding (one-year follow-up)	1 (1 early) (0.4%)	0 (0.0%)	n/a	n/a	1.00
Direct mortality	3 (1.2%)	1 (0.7%)	1.76	0.18–17.13	1.00
Reintervention (one-year follow-up)	0 (0.0%)	0 (0.0%)	n/a	n/a	1.00

## Discussion

### Changes of exclusion criteria for endovascular treatment

Comparison of the recent group of treated ruptured intracranial aneurysms with the earlier group showed a decrease of cases with aneurysm neck configuration which had led to an endovascular treatment exclusion. Dome-neck ratio < 1.5 and absolute neck width as a reason for exclusion decreased significantly from 21.1% to 9.7% (p = 0.0037), respectively 22.4% to 6.3% (p < 0.0001). Far distal aneurysms (14.2% to 38.2%; p < 0.0001) and the presence of an intracranial hematoma (8.9% to 20.1%; p = 0.0015) were the actual top reasons for microsurgical clipping in our study. Besides the standard coiling procedure many new endovascular occlusion techniques and devices like stent-assisted coiling procedures, WEB-devices and flow-diverters have been developed, which allow the treatment of aneurysms with unfavourable neck configuration [11–17]. Moreover, the ISAT-study resulted in a preference of the endovascular procedure for occlusion of ruptured intracranial aneurysms if both procedures (clipping and coiling) were possible [4–6]. O'Kelly et al. reported an increasing proportion of aneurysm treatment by coiling over time, but stated that endovascular treatment was associated with a significantly increased hazard of death or SAH readmission (hazard ratio 1.25; 95% CI 1.00–1.55; p = 0.04) [18]. Nevertheless, ISAT had a deep impact on the evaluation of the treatment procedure between the specialists of neurosurgery and neuroradiology and changed the decision making process in the group of 2006–2009 and 2012 to 2014 towards a higher ratio of endovascular treatment and less exclusion because of unfavourable neck configuration.

### Symptomatic ischemic stroke

The significantly lower absolute rate of symptomatic ischemic stroke (endovascular treatment 15.7% versus microsurgical clipping 5.3%) and permanent symptomatic ischemic stroke (endovascular treatment 13.9% versus microsurgical clipping 4.5%) in the microsurgical group of 1997–2003 persisted in the group of 2006–2009 and 2012–2014 (endovascular treatment 19.7% versus microsurgical clipping 9.0%, respectively endovascular treatment 15.3% versus microsurgical clipping 7.6%) (Tables 4 and 5). In total, an increase of the rate of symptomatic ischemic stroke was observed, although not significant. The procedure-related cerebral infarction rate of both groups were comparable with the results of Hoh et al (surgical group 11.4% versus endovascular group 21%) [19]. Some publications reported an association between new endovascular devices and symptomatic ischemic stroke or at least thrombembolic events [13, 15, 17]. The WEBCAST study observed thromboembolic events in 17.6% (9 of 51), but a permanent deficit was seen in only 1 patient (2.0%) [13]. Piotin et al. examined clinical and angiographic outcomes of 1137 consecutive patients coiled with and without stent-assisted coiling and detected a high permanent neurological procedure-related complication which occurred in 7.4% [15]. Van Rooij et al. even noticed that the safety and efficacy profile of flow diversion should discourage the use of these devices in aneurysms that can be treated with other techniques. In this collective 3 out of 11 patients treated with flow diverters suffered from ischaemia. Two died and one suffered from permanent neurological deficits [17].

In order to ensure a more balanced comparison of the endovascular coiling modality over time, we excluded the small subgroup of cases of aneurysmal SAH treated by Flow-Diverters, WEB-Devices and Stent-assisted Coiling procedures in the recent collective.

**Fig 1:**
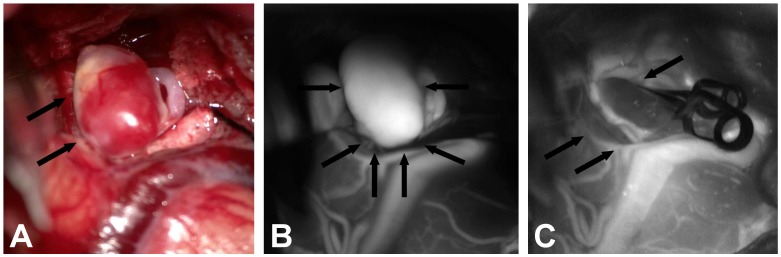
Microscope-based indocyanine green video angiography supporting the microsurgical occlusion of a ruptured middle cerebral artery aneurysm, which has been tested but is not used as a routine intraoperative device so far. (A): Intraoperative illustration of the middle cerebral artery aneurysm (arrows). (B): Video angiography showing the perfusion of the untreated aneurysm (arrows). (C): After clipping no perfusion of the aneurysm is detectable any more (arrows).

Reasons for the increase of symptomatic ischemic strokes in the microsurgical procedure arm might include a lack of routine of the treating specialists due to decreasing numbers of microsurgical clipping. New supporting devices for the microsurgical clipping procedure like microscope-based indocyanine green video angiography have been developed (Fig 1). This provides a clear real-time image with information about aneurysm configuration and intracranial artery pathways [20]. In future this device might lead to better outcome rates in the microsurgical procedure arm, but currently this device is not used routinely in all neurological centers. Nevertheless, there was no significant difference regarding the rate of symptomatic ischemic stroke in the direct comparison of the chronological groups (Tables 6 and 7).

### Occlusion-rate and reinterventions

The microsurgical clipping procedure was significantly superior regarding the occlusion of ruptured aneurysms in the recent and in the older group, but initial occlusion status in the microsurgical clipping group has been evaluated in most cases by the treating surgeon and not by angiographic control as in the endovascular group. In the follow-up regarding complete occlusion the rate of angiographic controls in the endovascular group (88.7% earlier group; 83.2% recent group) was higher than in the microsurgical clipping group (34.6% earlier group; 23.6% recent group). Initial subtotal occlusion was stated in 1.6% percent of the microsurgical cases versus 19.1% of the endovascular cases in the older group (OR 14.31; 95% CI 4.80–42.65; p < 0.0001). In the recent group there were 2.1% in microsurgical clipping group and 14.6% in the endovascular procedure group (OR 8.03; 95% CI 2.33–27.71; p = 0.00012). The initial incomplete occlusion rate remained nearly unchanged in the endovascular group (7.0% versus 6.6%) (Table 6) and in the microsurgical clipping group (0.0% versus 0.7%). The significantly lower rate of initial incomplete occlusion in the microsurgical group remained stable (older group p < 0.0001; recent group p = 0.0089). The complete occlusion rate in the follow-up was not improved significantly in the endovascular group (63.7% versus 64.0%), but the follow-up interval was only 13.1 months in the recent group, as compared with 22.3 months in the early group. Compared with ISAT and BRAT (Barrow Ruptured Aneurysm Trial) the complete obliteration rate of the ruptured aneurysms in the endovascular group seems quite similar [6, 21]. In BRAT endovascular treatment reached a complete obliteration of the ruptured aneurysms in 57.9% initially, in 52.2% after three years and in 47.9% after six years [21, 22]. In ISAT the complete occlusion rate in the endovascular group (discharge until over 2 years) was 66% of the treated aneurysms and therefore was almost the same as in our groups (63.7% and 64.0%, respectively) [6]. Large studies dealing with stent-assisted coiling report even better complete occlusion rates and are indicative of an improved complete occlusion rate over time [15, 16].

Very interesting is the significant decreasing rate of reinterventions in the recent endovascular group (13.9% versus 5.1%). Still, there was no case of reintervention in the microsurgical group, which remained significantly better in comparison to the endovascular group (older group: p < 0.0001; recent group: p = 0.0060). In the microsurgical clipping group the complete occlusion rate decreased significantly from 98.8% to 88.2% (p = 0.023). However, compared to the earlier group (85 of 246; 34.6%), in the recent group only 34 follow-up angiographic controls had been performed (23.6%). The significantly better complete occlusion rate in the follow-up of the microsurgical clipping group versus the endovascular group persisted (older group: 98.8%; p < 0.0001; recent group: 88.2%; p = 0.0071). Moreover, the complete occlusion rate still is comparable with the results of ISAT (82%) and BRAT (85.1%, 87.1% after 3 years and 95.7% after 6 years) [6, 21, 22].

### Rebleeding

The rebleeding rate was significantly higher in the endovascular group compared to the microsurgical group in the older collective (6.1% versus 0.4%; p = 0.0017) (Table 4). Interestingly the rebleeding rate in the endovascular group dropped to 2.2% and was no longer significantly different compared to the microsurgical clipping group (endovascular treatment 2.2% versus microsurgical treatment 0.0%, p = 0.11) (Table 5). In comparison to other studies like ISAT (2.6% endovascular rebeleeding rate) and CARAT (3.4% endovascular rebleeding rate) the rebleeding rate was initially higher (6.1%) but decreased under the values of these studies in time (2.2%) [4, 23]. Also, Fleming et al. stated that the periprocedural rebleeding rate may be improving over time regarding endovascular treatment [24]. The rebleeding rate in the microsurgical clipping arm is exceptionally low compared to ISAT (0.93%) and CARAT (1.3%) [4, 23].

### Direct mortality

Direct mortality was not significantly different between the two groups (endovascular treatment 2.6% versus microsurgical clipping 1.2%; p = 0.39) and remained stable (endovascular group: 2.9%—microsurgical clipping group: 0.7%; p = 0.20). Park et al. describe a direct endovascular mortality rate of 7.6% in ruptured aneurysms [25], while Zheng et al. report a mortality rate of 0.85% [26]. A direct mortality rate of 0.8% was stated for the microsurgical clipping procedure in the treatment of non-ruptured aneurysms [27]. De Vries et al. found a combined procedural persistent neurological morbidity and mortality after endovascular treatment and open surgical clipping of 2.1% and 1.4%, respectively [28]. Thus, in our collective the endovascular procedure and microsurgical clipping are save interventions with a low direct mortality rate which does not differ significantly over time and which is comparable with the direct mortality rates of other authors.

### Limitations

The location of aneurysms treated in the endovascular and microsurgical groups were different (Tables 2 and 3). Endovascular treatment focused on the posterior circulation, anterior communicating, anterior cerebral, and internal carotid aneurysms (in the recent group). Middle cerebral artery (MCA) aneurysms and internal carotid (ICA) aneurysms (in the earlier group) were usually treated by microsurgical clipping. But regarding the initial clinical status (WFNS grading) of the patients there was a balance concerning the patients with WFNS°IV-V in the recent and in the earlier group and also for WFNS°I-II and III in the recent group (Tables 2 and 3).

In contrast to the endovascular arm, the rate of post-operative angiographic imaging for the evaluation of occlusion success was low in the clipping arm. Furthermore, initial occlusion rates for microsurgical clipping were largely based on post-operative surgeon reports, which are always subject to bias. These circumstances certainly increased the detection of small residuals found post-treatment in the endovascular arm. Moreover, we presented non-randomized data with short-term results without clear outcome data like mRS and GOS in this study.

## Conclusion

Here, we present a prospective, non-randomized trial in which we analyzed the changes in the daily decision process regarding the procedure assignment and treatment of ruptured intracranial aneurysms by experts of neuroradiology and neurosurgery. The number of reinterventions at one-year follow-up due to refilled treated aneurysms significantly decreased in the endovascular group over time, but the significantly better occlusion- and reintervention-rate of the microsurgical group persisted. Rebleeding occurred less frequently in the endovascular group over time, although we can only present rates until the one-year follow–up, but no long term results. Endovascular treatment was chosen significantly more often over time. Accordingly, cost effectiveness analyses for endovascular versus microsurgical clipping could become attractive. Higher device costs of endovascular treatment might imply that this development will lead to higher financial expenditure [29]. However, Hoh et al. conducted a nation-wide cost-analysis in which an association of endovascular treatment and a significantly shorter length of stay and significantly lower total hospital charges in comparison to the microsurgical group were exhibited [30]. Hence, this development comes along with an economic as well as with a clinical improvement regarding the efficacy and safety of treatment of ruptured intracranial aneurysms.

## References

[1] de Rooij NK, Linn FH, van der Plas JA, Algra A, Rinkel GJ. Incidence of subarachnoid haemorrhage: a systematic review with emphasis on region, age, gender and time trends. J Neurol Neurosurg Psychiatry. 2007;78(12):1365–72. 10.1136/jnnp.2007.117655 17470467 PMC2095631

[2] Steiner T, Juvela S, Unterberg A, Jung C, Forsting M, Rinkel G, et al European Stroke Organization guidelines for the management of intracranial aneurysms and subarachnoid haemorrhage. Cerebrovasc Dis. 2013;35(2):93–112. 10.1159/000346087 23406828

[3] Connolly ES Jr., Rabinstein AA, Carhuapoma JR, Derdeyn CP, Dion J, Higashida RT, et al Guidelines for the management of aneurysmal subarachnoid hemorrhage: a guideline for healthcare professionals from the American Heart Association/american Stroke Association. Stroke. 2012;43(6):1711–37. 10.1161/STR.0b013e3182587839 22556195

[4] Molyneux A, Kerr R, International Subarachnoid Aneurysm Trial Collaborative G, Stratton I, Sandercock P, Clarke M, et al International Subarachnoid Aneurysm Trial (ISAT) of neurosurgical clipping versus endovascular coiling in 2143 patients with ruptured intracranial aneurysms: a randomized trial. J Stroke Cerebrovasc Dis. 2002;11(6):304–14. 10.1053/jscd.2002.130390 17903891

[5] Molyneux AJ, Kerr RS, Birks J, Ramzi N, Yarnold J, Sneade M, et al Risk of recurrent subarachnoid haemorrhage, death, or dependence and standardised mortality ratios after clipping or coiling of an intracranial aneurysm in the International Subarachnoid Aneurysm Trial (ISAT): long-term follow-up. Lancet Neurol. 2009;8(5):427–33. 10.1016/S1474-4422(09)70080-8 19329361 PMC2669592

[6] Molyneux AJ, Kerr RS, Yu LM, Clarke M, Sneade M, Yarnold JA, et al International subarachnoid aneurysm trial (ISAT) of neurosurgical clipping versus endovascular coiling in 2143 patients with ruptured intracranial aneurysms: a randomised comparison of effects on survival, dependency, seizures, rebleeding, subgroups, and aneurysm occlusion. Lancet. 2005;366(9488):809–17. 10.1016/S0140-6736(05)67214-5 16139655

[7] Ryttlefors M, Enblad P, Kerr RS, Molyneux AJ. International subarachnoid aneurysm trial of neurosurgical clipping versus endovascular coiling: subgroup analysis of 278 elderly patients. Stroke. 2008;39(10):2720–6. 10.1161/STROKEAHA.107.506030 18669898

[8] Hammer A, Steiner A, Kerry G, Ranaie G, Yakubov E, Lichtenstern D, et al Efficacy and Safety of Treatment of Ruptured Intracranial Aneurysms. World Neurosurg. 2016. 10.1016/j.wneu.2016.07.013 27423199

[9] Sprengers ME, Schaafsma J, van Rooij WJ, Sluzewski M, Rinkel GJ, Velthuis BK, et al Stability of intracranial aneurysms adequately occluded 6 months after coiling: a 3T MR angiography multicenter long-term follow-up study. AJNR Am J Neuroradiol. 2008;29(9):1768–74. 10.3174/ajnr.A1181 18583406 PMC8118778

[10] van Rooij WJ, Sluzewski M. Opinion: imaging follow-up after coiling of intracranial aneurysms. AJNR Am J Neuroradiol. 2009;30(9):1646–8. 10.3174/ajnr.A1673 19617448 PMC7051490

[11] Bozzetto Ambrosi P, Gory B, Sivan-Hoffmann R, Riva R, Signorelli F, Labeyrie PE, et al Endovascular treatment of bifurcation intracranial aneurysms with the WEB SL/SLS: 6-month clinical and angiographic results. Interv Neuroradiol. 2015;21(4):462–9. 10.1177/1591019915590083 26111987 PMC4757321

[12] de Paula Lucas C, Piotin M, Spelle L, Moret J. Stent-jack technique in stent-assisted coiling of wide-neck aneurysms. Neurosurgery. 2008;62(5 Suppl 2):ONS414–6; discussion ONS6-7. 10.1227/01.neu.0000326028.47090.5f 18596523

[13] Pierot L, Costalat V, Moret J, Szikora I, Klisch J, Herbreteau D, et al Safety and efficacy of aneurysm treatment with WEB: results of the WEBCAST study. J Neurosurg. 2015:1–7. 10.3171/2015.2.JNS142634 26381253

[14] Piotin M, Blanc R. Balloons and stents in the endovascular treatment of cerebral aneurysms: vascular anatomy remodeled. Front Neurol. 2014;5:41 10.3389/fneur.2014.00041 24782817 PMC3986530

[15] Piotin M, Blanc R, Spelle L, Mounayer C, Piantino R, Schmidt PJ, et al Stent-assisted coiling of intracranial aneurysms: clinical and angiographic results in 216 consecutive aneurysms. Stroke. 2010;41(1):110–5. 10.1161/STROKEAHA.109.558114 19959540

[16] Sedat J, Chau Y, Mondot L, Vargas J, Szapiro J, Lonjon M. Endovascular occlusion of intracranial wide-necked aneurysms with stenting (Neuroform) and coiling: mid-term and long-term results. Neuroradiology. 2009;51(6):401–9. 10.1007/s00234-009-0502-2 19241069

[17] van Rooij WJ, Bechan RS, Peluso JP, Sluzewski M. Endovascular treatment of intracranial aneurysms in the flow diverter era: frequency of use and results in a consecutive series of 550 treatments in a single centre. Interv Neuroradiol. 2014;20(4):428–35. 25207905 10.15274/INR-2014-10047 PMC4187438

[18] O'Kelly CJ, Kulkarni AV, Austin PC, Wallace MC, Urbach D. The impact of therapeutic modality on outcomes following repair of ruptured intracranial aneurysms: an administrative data analysis. Clinical article. J Neurosurg. 2010;113(4):795–801. 10.3171/2009.9.JNS081645 19852537

[19] Hoh BL, Curry WT Jr., Carter BS, Ogilvy CS. Computed tomographic demonstrated infarcts after surgical and endovascular treatment of aneurysmal subarachnoid hemorrhage. Acta Neurochir (Wien). 2004;146(11):1177–83. 15349755 10.1007/s00701-004-0349-6

[20] Raabe A, Nakaji P, Beck J, Kim LJ, Hsu FP, Kamerman JD, et al Prospective evaluation of surgical microscope-integrated intraoperative near-infrared indocyanine green videoangiography during aneurysm surgery. J Neurosurg. 2005;103(6):982–9. 10.3171/jns.2005.103.6.0982 16381184

[21] Spetzler RF, McDougall CG, Albuquerque FC, Zabramski JM, Hills NK, Partovi S, et al The Barrow Ruptured Aneurysm Trial: 3-year results. J Neurosurg. 2013;119(1):146–57. 10.3171/2013.3.JNS12683 23621600

[22] Spetzler RF, McDougall CG, Zabramski JM, Albuquerque FC, Hills NK, Russin JJ, et al The Barrow Ruptured Aneurysm Trial: 6-year results. J Neurosurg. 2015;123(3):609–17. 10.3171/2014.9.JNS141749 26115467

[23] Johnston SC, Dowd CF, Higashida RT, Lawton MT, Duckwiler GR, Gress DR, et al Predictors of rehemorrhage after treatment of ruptured intracranial aneurysms: the Cerebral Aneurysm Rerupture After Treatment (CARAT) study. Stroke. 2008;39(1):120–5. 10.1161/STROKEAHA.107.495747 18048860

[24] Fleming JB, Hoh BL, Simon SD, Welch BG, Mericle RA, Fargen KM, et al Rebleeding risk after treatment of ruptured intracranial aneurysms. J Neurosurg. 2011;114(6):1778–84. 10.3171/2011.1.JNS101232 21332293

[25] Park HK, Horowitz M, Jungreis C, Genevro J, Koebbe C, Levy E, et al Periprocedural morbidity and mortality associated with endovascular treatment of intracranial aneurysms. AJNR Am J Neuroradiol. 2005;26(3):506–14. 15760857 PMC7976477

[26] Zheng Y, Liu Y, Leng B, Xu F, Tian Y. Periprocedural complications associated with endovascular treatment of intracranial aneurysms in 1764 cases. J Neurointerv Surg. 2015. 10.1136/neurintsurg-2014-011459 25564539

[27] Krisht AF, Gomez J, Partington S. Outcome of surgical clipping of unruptured aneurysms as it compares with a 10-year nonclipping survival period. Neurosurgery. 2006;58(2):207–16; discussion -16. 10.1227/01.NEU.0000194638.61073.FC 16462473

[28] de Vries J, Boogaarts HD. Treatment of patients with ruptured aneurysm by neurosurgeons that perform both open surgical and endovascular techniques is safe and effective: results of a single centre in Europe. Acta Neurochir (Wien). 2014;156(7):1259–66; discussion 66. 24789710 10.1007/s00701-014-2101-1

[29] Hoh BL, Chi YY, Dermott MA, Lipori PJ, Lewis SB. The effect of coiling versus clipping of ruptured and unruptured cerebral aneurysms on length of stay, hospital cost, hospital reimbursement, and surgeon reimbursement at the university of Florida. Neurosurgery. 2009;64(4):614–9; discussion 9–21. 10.1227/01.NEU.0000340784.75352.A4 19197221

[30] Hoh BL, Chi YY, Lawson MF, Mocco J, Barker FG 2nd. Length of stay and total hospital charges of clipping versus coiling for ruptured and unruptured adult cerebral aneurysms in the Nationwide Inpatient Sample database 2002 to 2006. Stroke. 2010;41(2):337–42. 10.1161/STROKEAHA.109.569269 20044522

